# Pharmacokinetics and biodistribution of the cyclin-dependent kinase inhibitor -CR8- in mice

**DOI:** 10.1186/2050-6511-14-50

**Published:** 2013-09-30

**Authors:** Hatem Sallam, Ibrahim El-Serafi, Laurent Meijer, Moustapha Hassan

**Affiliations:** 1 Experimental Cancer Medicine (ECM), Laboratory Medicine, Karolinska Institutet, 141 86, Stockholm, Sweden; 2 C.N.R.S., ‘Protein Phosphorylation and Human Disease’ Group, C.N.R.S., Station Biologique, B.P. 74, 29682Roscoff cedex, Bretagne, France; 3 Experimental Cancer Medicine (ECM), Clinical Research Center, Novum, Karolinska University Hospital-Huddinge, 141 86, Stockholm, Sweden; 4 ECM, KFC, Karolinska University Hospital, 141 86, Stockholm, Sweden

**Keywords:** Roscovitine, CR8, Pharmacokinetics, Cyclin-dependent kinase inhibitors, Kinase inhibitors, Biodistribution, HPLC

## Abstract

**Background:**

CR8 is a second generation inhibitor of cyclin-dependent kinases derived from roscovitine. CR8 was shown to be 50–100 fold more potent than roscovitine in inducing apoptosis in different tumor cell lines. In the present investigation, we have established an analytical method for the quantification of CR8 in biological samples and evaluated its bioavailability, biodistribution and pharmacokinetics in mice.

**Methods:**

A liquid chromatography method utilizing UV-detection was used for the determination of CR8. CR8 was administered either orally (100 mg/kg) or i.v. (50 mg/kg) and the animals were sacrificed at different time points. Blood samples and organs were collected, after which the pharmacokinetic parameters were calculated for plasma and organs.

**Results:**

CR8 was eluted at 5 minutes in the high performance liquid chromatography system used. The LLOQ detection was 0.10 μg/ml and linearity was observed within the 0.10-10 μg/ml range (r^2^ > 0.998). The accuracy and precision were >86%, while the recovery from plasma was >95%. CR8 was stable for 2 months at room temperature in both solution and plasma. CR8 pharmacokinetics was fitted to a two-compartment open model after oral administration and to a one compartment model after i.v. injection. The elimination half-life was about 3 hours. Organ exposure to CR8 (expressed as % AUC organ vs. AUC plasma) was highest in liver (205%), adipose tissue (188%) and kidney (150%) and low in bone marrow (30%) and brain (15%) as compared to plasma. The oral bioavailability of CR8 was found to be essentially 100%.

**Conclusions:**

We have developed a rapid and simple method for the analysis of CR8. CR8 pharmacokinetics pattern showed 100% bioavailability, long half-life and limited distribution to brain and bone marrow, which may allow systemic exposure higher than the IC_50_ reported for cell death in tumor cell lines. CR8 displays favorable pharmacological properties and is therefore a good candidate for future clinical studies.

## Background

**Figure 1:**
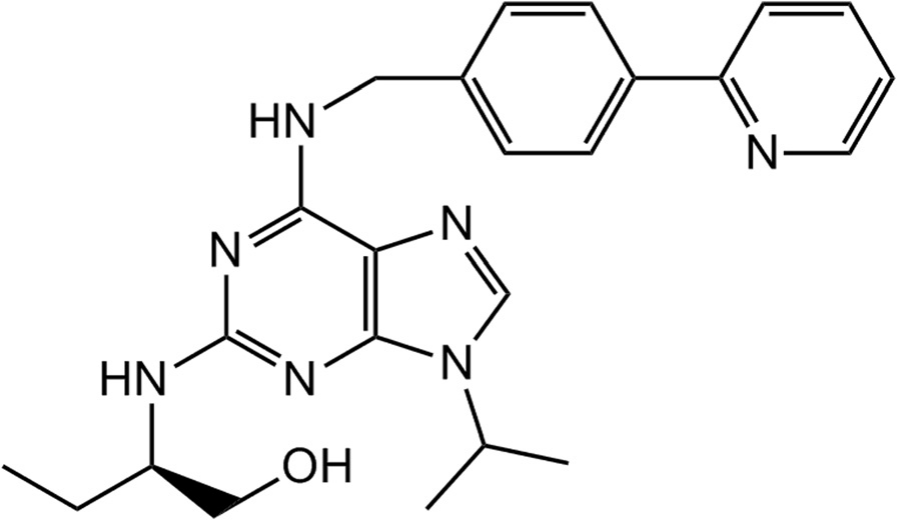
**CR8:** 2-(*R***)-(1-Ethyl-2-hydroxyethylamino)-6-(4-(2-pyridyl)benzyl)-9-isopropylpurine (CR8).**

Roscovitine is a selective cyclin-dependent kinase (CDK) 1, 2, 5, 7, and 9 inhibitor [1-4] shown to be effective against several tumors. At present, roscovitine is undergoing Phase II clinical trials in cancer patients (non-small cell lung cancer, nasopharyngeal cancer). The drug has limited side effects and toxicity compared to conventional chemotherapy [5]. However, roscovitine is shown to have a short elimination half-life and rapid metabolism, which leads to suboptimal exposure in clinical trials. Recently, 2-(*R*)-(1-Ethyl-2-hydroxyethylamino)-6-(4-(2-pyridyl)benzyl)-9-isopropylpurine (CR8; Figure 1) was introduced as a second generation cyclin-dependent kinase inhibitor derived from roscovitine [6]. CR8 is shown to be about 50-fold more potent compared to roscovitine in inducing apoptosis in different tumor cell lines including ALL and CLL [7].

Several *in-vivo* and *in-vitro* studies have reported promising results due to the high efficacy of CR8. Bukanov et al. show CR8 to be effective against autosomal dominant polycystic kidney disease by blocking the renal and hepatic cystogenesis in mice [8]. Another study reports that CR8 can induce apoptotic tumor cell death in the neuroblastoma cell line, which is one of the most frequent solid tumors in children [9]. CR8 is also proven to be a neuro-protector in experimental traumatic brain injury and has significantly reduced the lesion volume in rats subjected to moderate spinal cord contusion injury [10,11]. Moreover, CR8 is shown to increase the number of surviving neurons after spinal cord injury and to decrease the posttraumatic elevation of biochemical markers of apoptosis in an animal model [12].

Generally, the final effects of drugs *in-vivo* are influenced by many factors, such as systemic exposure, distribution in various organs, metabolism, lipophilicity, and protein binding. Bettayeb et al. show the optimal concentrations of CR8 needed to obtain a desirable pharmacological effect *in-vitro* in different cell lines to be about 0.7 μM [7], which is far below that reported for roscovitine (14.6 μM) [4].

Despite these results obtained in cell cultures, the pharmacokinetic profile of CR8 needs to be analyzed prior to further preclinical and possibly clinical investigations. In the present investigation we have established and validated an analytical method for the quantitative determination of CR8 according to standard bioanalytical guidelines. We have also investigated the pharmacokinetics profile and the tissue distribution of CR8 in mice. The present results are encouraging in terms of further development of CR8 as a drug candidate.

## Methods

### Chemicals and reagents

Tetrahydrofuran and methanol of high performance liquid chromatography (HPLC) grade were obtained from Merck (Darmstadt, Germany), Tween20 and dimethyl sulfoxide (DMSO) from Sigma-Aldrich (Stockholm, Sweden). All other reagents and solvents were of HPLC analytical grade. S-CR8 was dissolved in DMSO as a 75 mg/ml stock solution and was stored at −20°C. Serial dilutions were prepared from the main stock for the calibration curves, as quality control samples and for administration to mice. (R)-roscovitine and (S)-CR8 were synthesized as described previously [13].

### Instrumentation and chromatographic conditions

The HPLC system consisted of a Gilson 234 auto-injector equipped with a 100 μl loop, an LKB 2150 pump (Pharmacia inc., Sweden), an “LDC analytical spectro-monitor 3200” UV-detector (USA) and a CSW 32 chromatography station integrator. Separation was performed on a Zorbax SB-CN column (3.5 μm × 4.6 mm × 150 mm) from Agilent (USA), while the column was maintained at room temperature during analysis.

The mobile phase consisted of tetrahydrofuran: 25 mM phosphate buffer pH = 2.6, ionic strength = 0.022 (20:80, v/v). Flow rate was set at 0.9 ml/min, running time at 8 min for standard curve and quality controls and at 15 min for animal samples to elute all the metabolites. Injection volume was 50 μl and the UV wavelength used was 305 nm. Standard curves were prepared from spiked pooled human plasma, and were linear in the range of 0.1 - 10 μg/ml.

### Standard solutions and controls

Pooled heparin plasma from healthy donors was obtained from the Blood Transfusion Center, Karolinska University Hospital. Serial standard dilutions in 50 mM Hydrochloric acid were prepared from the stocks for the calibration curves and for the quality controls.

Working standard solutions of CR8 in plasma at 10, 8, 6, 5, 4, 3, 2, 1, 0.5, 0.25 and 0.1 μg/ml were prepared by adding 50 μl from the standard stocks to 950 μl plasma to reach 1 ml containing the final concentrations. The calibrators were prepared on the day of each run.

Four quality control samples at concentrations of 0.75, 1.5, 3.5 and 7.0 μg/ml were also prepared in triplicate and stored at room temperature, +4°C and −20°C for stability testing.

The quality control samples were repeated using mouse plasma in triplicate using the same concentrations as in human plasma.

### Sample preparation from plasma

70 μl plasma was added to 140 μl methanol and vortexed for 15 sec. Samples were centrifuged at 10000 g for 10 minutes, and 50 μl from the supernatant were injected into the HPLC system.

### Bioanalytical method validation

#### Selectivity

A selective method should involve no interference from endogenous compounds, metabolites and/or different degradation products related to the drug [14]. We confirmed that our method involved no such interference by running blank biological samples from untreated mice and mice receiving only the drug vehicle [15].

#### Calibration curve

A calibration curve was constructed from eight standard points of CR8 at concentrations ranging from 0.1 - 10 μg/ml, including lower limit of quantification (LLOQ). The peak areas of CR8 were plotted against the concentrations. Linearity was assessed by a weighted least-squares regression analysis and a correlation coefficient (*r*^2^) of 0.99 or better was deemed acceptable. The LLOQ was set as the lowest measurable concentration with acceptable accuracy and precision not more than 20% of the expected values [16].

#### Precision and accuracy

The precision of the method was defined as the percent relative standard deviation (% SD) calculated from triplicate measurements [(standard deviation)/mean value] × 100. The accuracy was defined as the percent relative error (% E) of the mean of the replicate measurements in relation to the theoretical values [(measured value - nominal value)/nominal value] × 100 [16,17]. Precision and accuracy were determined by analyzing quality control samples (QCs) prepared at four concentrations (0.75, 1.5, 3.5 and 7.0 μg/ml) in pentaplicate on three consecutive days [18].

#### Recovery

Absolute recovery is defined as percentage of reference compound that is measured to the exact amount of compound added to a blank buffer [15]. Recovery was computed by comparing responses in triplicate of extracted low, middle and high QCs (0.75, 1.5 and 7.0 μg/ml, respectively) samples with those of extracted solvent to which CR8 has been added at the same nominal concentration [14].

Relative recovery is defined as percentage of the amount of drug measured from the plasma compared to the amount measured from pure solvent e.g. 50 mM hydrochloric acid (HCl) [15]. The low, medium and high controls (0.75, 1.5 and 7.0 μg/ml, respectively) were prepared in pooled plasma and in 50 mM HCl. Samples were run in triplicate and recovery was calculated as the percentage of (plasma peak area/methanol peak area) × 100.

#### Stability

The stability of CR8 was determined in plasma samples using concentrations of 0.75, 1.5, 3.5 and 7.0 μg/ml (in triplicate). The samples were kept at room temperature, +4°C and −20°C and analyzed twice weekly for 2 months. Peak areas of all the samples were compared to peak areas obtained at time zero.

### Animals

All experiments in this study were approved by the Stockholm Southern Ethics Committee for Animal Research and were conducted in accordance with the Animal Protection Law, the Animal Protection Regulation and the Regulation for the Swedish National Board for Laboratory Animals.

Female BALB/c mice 8–10 weeks old and weighing 18–21 g were obtained from Scanbur, Sollentuna, Sweden. The mice were allowed to acclimatize to their surroundings for one week before starting treatment. The mice were fed pelleted food and water *ad libitum*.

CR8 was dissolved in DMSO: 50 mM HCl (10:90 v/v). The first group of mice received i.v. doses of CR8 (50 mg/kg body weight), the second received oral doses of CR8 (100 mg/kg body weight) and three control mice received vehicle alone. Three mice were sacrificed at each time point and blood samples were collected by cardiac puncture into heparinized tubes at 10, 20, 30, 60 min, 2, 4, 6, 8 h post administration. Blood samples were centrifuged immediately after collection at 3000 g for 5 min at 4°C and the plasma was prepared as mentioned previously. Supernatants were collected and all samples were frozen at −20°C directly after preparation until further analysis by HPLC. Other organs including liver, spleen, kidneys, brain, adipose tissue and lungs were removed, washed and snap frozen immediately in liquid nitrogen. Bone marrow was flushed from both femurs and mononuclear cells were counted and frozen. The samples were stored at −20°C to avoid any metabolic activity.

### Tissue sample preparation

Samples from different organs were homogenized by probe sonication in sodium perborate (PBS) (1:3 – 1:5 w:v) for 2 min and vortexed for 1 min. 100 μl of the homogenate was added to 200 μl methanol, vortexed for 1 min and centrifuged at 10000 g for 10 min. 50 μl of the supernatant were injected into the HPLC system.

Femurs were removed and cleaned; bone marrow was flushed with 0.3 ml of PBS and single cell suspension was prepared by gentle flushing through needle and syringe.

Nucleated cells were counted using Türk solution and bone marrow was stored at −20°C until assay.

Quality control samples were run together with the corresponding mouse samples in duplicate.

### Pharmacokinetics

Parameters including distribution volume of the central compartment, elimination rate constant, plasma maximum concentration and micro constants were estimated. Clearance (Cl) and distribution volume at the steady state were calculated from the primary parameters. The plasma concentration versus time curves (AUC) were calculated from the model derived parameters, and the elimination half-lives were calculated from the slope of the terminal elimination phase. Bone marrow data were derived after adjustment of concentrations to 10^9^ cells (= approx. 1 g of tissue). The pharmacokinetic modeling was performed using WinNonlin version 5.2 (Pharsight, USA).

Oral bioavailability (F) defined as the fraction of the oral administered dose of CR8 that reaches the systemic circulation was calculated according to the following formula:

$$F=\frac{{\left[\mathrm{AUC}\right]}_{\mathrm{po}}*\;\mathrm{dos}e_{\mathrm{IV}}}{{\left[\mathrm{AUC}\right]}_{\mathrm{IV}}*\;\mathrm{dos}e_{\mathrm{po}}}$$

### Calculations and statistics

The peak areas of CR8 were plotted versus the corresponding nominal concentrations of the standards, and the standard curve was calculated by linear regression. CR8 concentrations in mouse plasma, organs and quality control samples were calculated from the resulting curves. All values are presented as mean ± S.D.

## Results

### Analysis of CR8

A precise and accurate method was developed to detect the drug in plasma and tissue samples in order to investigate the pharmacokinetics of CR8. CR8 is a lipophilic compound; however, due to the presence of the 2-pyridyl ring attached to the benzyl ring, its lipophilicity is lower than that of the parent analogue roscovitine. DMSO was found to be a suitable solvent for very high concentrations of CR8, and 50 mM HCl was found to be a suitable solvent for standard concentrations. Protein precipitation is the simplest approach for removing most proteins from biological samples. Methanol was shown to be better than acetonitrile in deproteinizing the plasma and extracting CR8 due to lower number of interfering peaks.

#### UV absorbance

UV absorbance was determined by running a UV-IVIS spectrum scan of CR8 dissolved in 50 mM HCl (10 μg/ml). No interference in the absorption range was detected. The scan revealed good absorption in the UV 210 nm - 310 nm wavelength ranges, and 305 nm was selected as the best measurement wavelength to ensure good selectivity and good sensitivity with a wide range of linear response for construction of the standard curves.

#### Chromatograms and specificity

**Figure 2:**
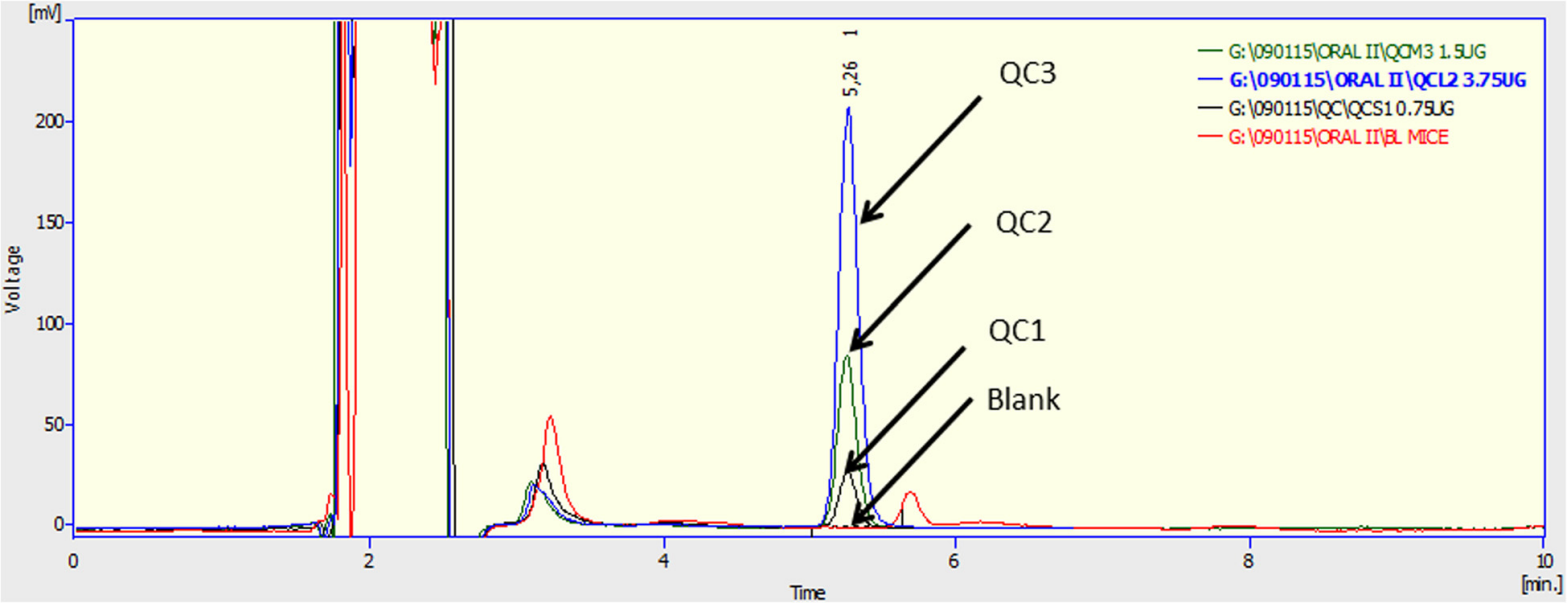
Quality control samples in human plasma: HPLC chromatograms of blank samples and plasma containing CR8 (QCs) at concentrations of 1.5, 3.75 and 10.75 μg/mL.

**Figure 3:**
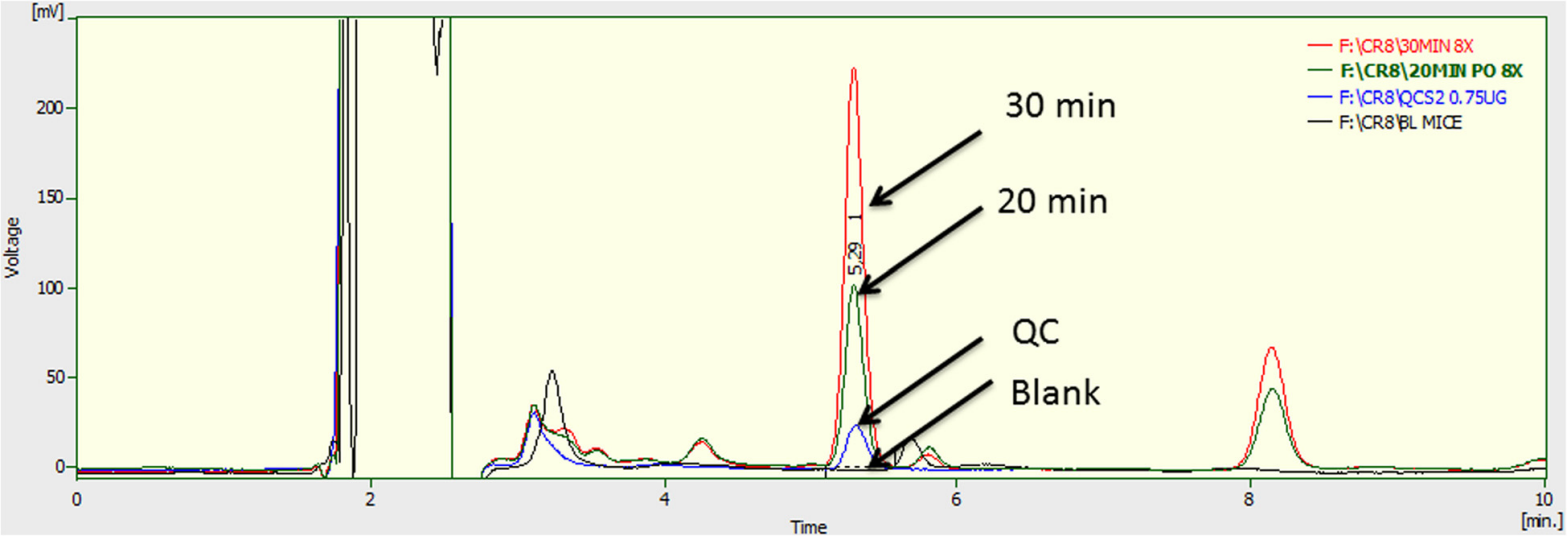
HPLC chromatograms of mouse plasma: The chromatograms contain blank sample, QC (0.75 μg/mL) and CR8 at 20 and 30 min after oral administration.

The CR8 retention time was around 5.26 ± 0.3 min. Selectivity was confirmed by the absence of significant interfering peaks from endogenous compounds in the blank plasma at this retention time (Figure 2). Also, no interference was found from endogenous compounds in the mouse plasma and tissue samples compared to control samples (Figure 3).

#### Linearity and limit of quantification

The calibration curve was linear within the ranges of 0.1 μg/ml - 10 μg/ml, and had a correlation coefficient (r^2^) ≥ 0.9987 (n = 6). The lower limit of quantification for CR8 was 0.1 μg/ml in human plasma. The precision and accuracy at LLOQ were 3.7 and 13.3%, respectively.

#### Accuracy and precision

The precision and accuracy between and within batch were always below 13% from the nominal values in all cases. Precision and accuracy fulfilled the standard guiding principles for validation of bioanalytical methods, i.e. < 15% for QCs both between batches and within batches and < 20% for LLOQ [14,16,17,19,20]. No differences were observed between mouse and human plasma concerning concentrations in QCs (Table 1).

**Table 1:** Precision and accuracy of the CR8 analysis (n = 3-5 each)

**Concentration (μg/ml)**	**Precision (%)**			**Accuracy (%)**		
*Between batch*						
**0.1** (LLOQ)	3.7			−13.3		
**0.75** (QC1)	7.7			−2.0		
**1.5** (QC2)	6.9			−3.8		
**3.5** (QC3)	4.9			−4.4		
**7.0** (QC4)	3.3			−5.3		
*Within batch*	*Batch1*	*Batch2*	*Batch3*	*Batch1*	*Batch2*	*Batch3*
**0.1** (LLOQ)	4.2	3.4	4.8	−16	−15.4	−8.5
**0.75** (QC1)	1.4	1.8	1.9	5.5	−12.9	−1.2
**1.5** (QC2)	1.5	1.7	0.8	4.3	−6.7	3.0
**3.5** (QC3)	0.2	0.6	1.1	−9.1	−9.9	−0.6
**7.0** (QC4)	0.8	2	1.3	−3.5	−7.4	−4.1

#### Recovery

The absolute recovery of CR8 in plasma was 97 ± 6%, 95 ± 5.0% and 98 ± 4% at the concentrations of 0.75, 1.5 and 7.0 μg/ml, respectively, while the relative recoveries of the drug for the same concentrations in plasma compared to that obtained from 50 mM HCl were 99 ±3, 103 ± 2 and 96 + 5%, respectively. The recovery from plasma was almost completely with low variability. Consequently, plasma could be a suitable matrix for the regular calibrations needed to analyze patient/animal materials in future *in-vivo* experiments [21].

#### Stability

The drug proved to be highly stable after 2 months at room temperature, +4°C or −20°C, with no significant decrease in the concentration of the QC samples (stability found to be approximately 97 ± 3.5%).

#### CR8 Metabolites

**Figure 4:**
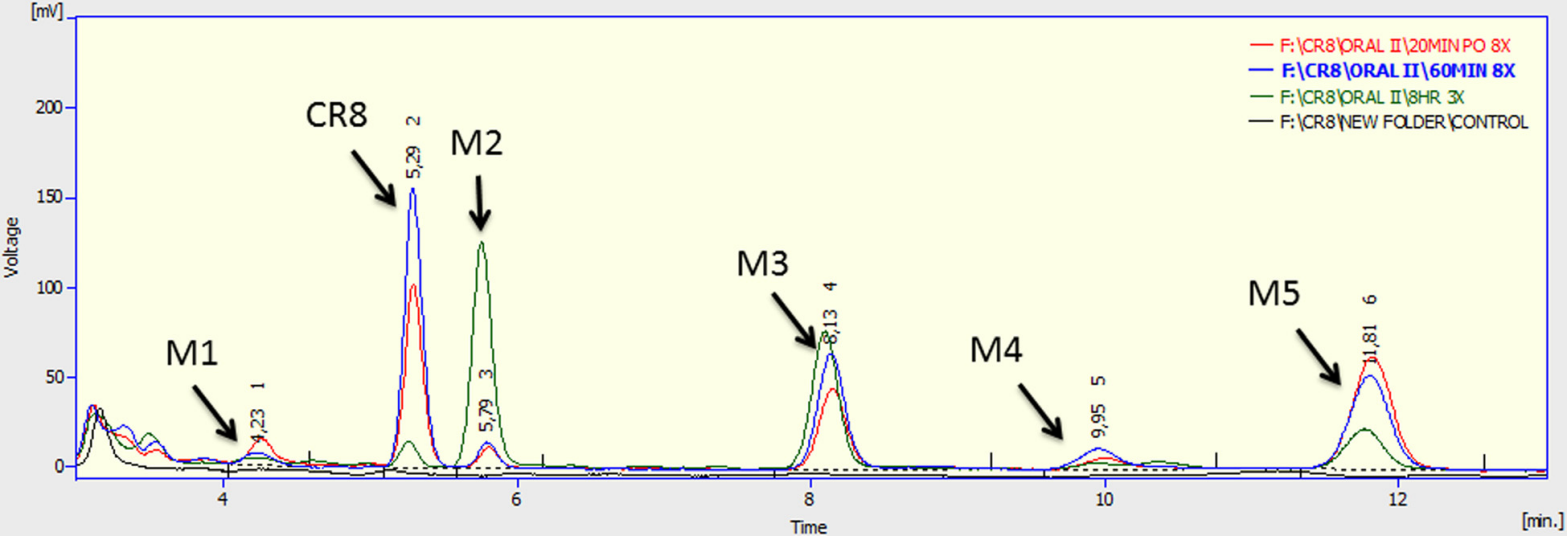
**HPLC chromatograms of CR8 and its metabolites.** Chromatograms showing CR8 and its five metabolites at different time points after oral administration, (red) 20 minutes post administration, (blue) 60 minutes post administration, (green) 8 hours post administration, (black) blank.

Five CR8 metabolites were separated and found to absorb UV at the same wavelength as CR8. These metabolites were named M1-M5 and appeared in the chromatogram in the following order: M1 at 4.23 minutes, M2 at 5.79 minutes, M3 at 8.13 minutes, M4 at 9.95 minutes and M5 at 11.81 minutes (Figure 4). The first and fifth metabolites (M1 & M5) appeared rapidly with maximum concentration at 20 minutes and slowly disappeared until 8 hours. The metabolite M4 increased by time to reach maximum concentration at 60 minutes then disappeared. The metabolites M2 and M3 increased and accumulated with time until 8 hours post administration (Figure 4).

### Pharmacokinetics of CR8

**Figure 5:**
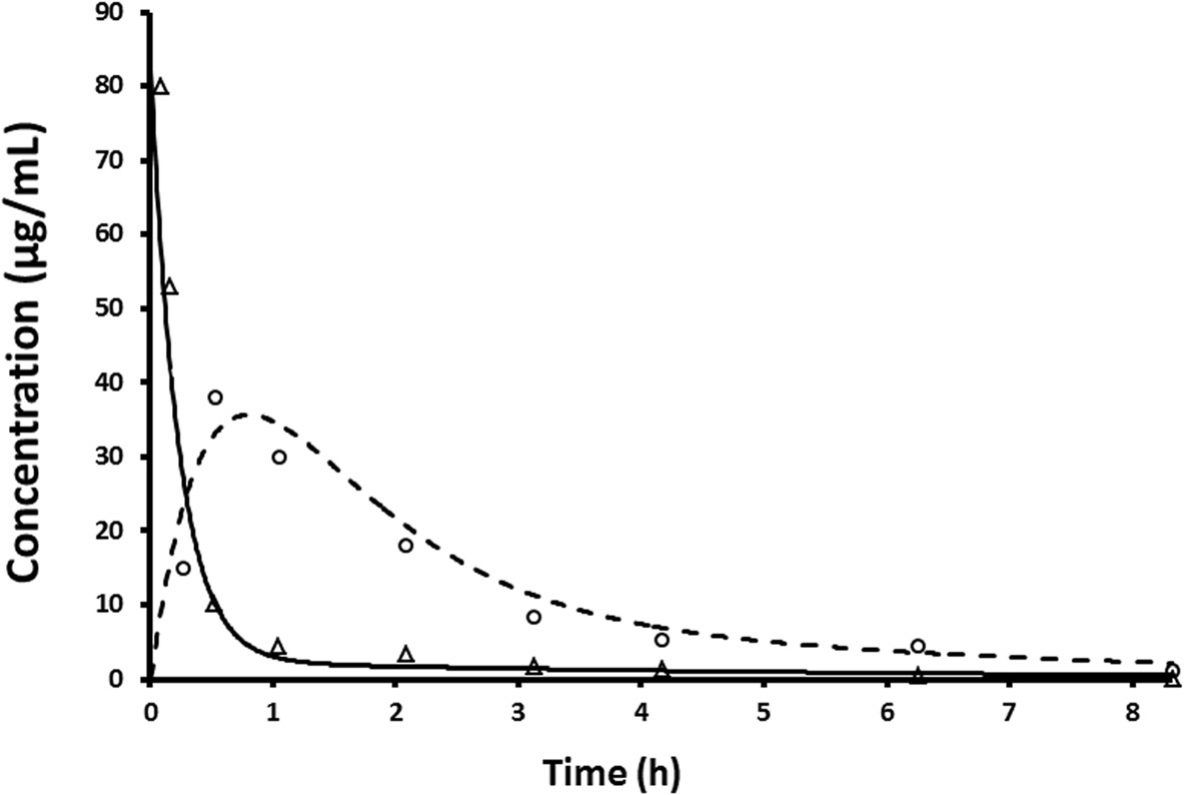
**Time–plasma concentration curve after PO and IV CR8 to the mouse.** Plasma time concentration curve after the oral administration of 100 mg/kg CR8 where the dashed line is the estimated two compartment model while the open circles are the observed concentrations. The solid line is the estimated one compartment model after the iv administration of 50 mg/kg CR8, while open triangles represent the measured concentrations.

To conduct the CR8 pharmacokinetic studies, a simple HPLC-UV method for the determination of CR8 in human plasma was developed and validated. The pharmacokinetics of CR8 were then investigated in mice and found to fit a two-compartment open model after oral administration and a one compartment bolus model after i.v. administration (Figure 5) using Gauss-Newton criteria.

The CR8 AUC was about 100 μg.hr/ml when 100 mg/kg was administered orally and about 45 μg.hr/ml when 50 mg/kg was administered intravenously. However, the estimated C_max_ was over 100 μg/ml after i.v. administration, which is 4 fold higher compared to that observed after oral administration (Table 2).

**Table 2:** PK parameters of CR8 following single oral administration at 100 mg/kg and i.v. bolus administration of 50 mg/kg

**PK parameters**	**Oral**	**i.v.**
AUC (h.μg/ml)	96.34 ± 10.53	44.35 ± 3.3
Tα (h)	1.3 ± 0.43	0.11 ± 0.01
Tβ (h)	3.1 ± 0.35	2.95 ± 0.11
C_max_ (μg/ml)	34.3 ± 1.94	163.9 ± 0.25
Vss (ml)	21 ± 3	44 ± 4
CL (ml/h)	21 ± 2	23 ± 2
Bioavailability	(96.43*50)/(44.35*100)*100 = 108%	

AUC: Area under time plasma concentration curve.

Tα: First elimination half-life.

Tβ: Terminal elimination half-life.

C_max_: Maximum reached concentration.

Vss: Distribution volume at steady state.

CL: Clearance.

Absorption and distribution were rapid and the maximum concentrations were found at 30 min. Most importantly, the elimination half-life was estimated to be about 3 hours.

Exposure to concentrations much higher than the reported average IC_50_ value (0.7 μM) [7] was found to last more than 8–10 hours.

The oral bioavailability of CR8 was calculated to be 108 ± 9%.

#### Biodistribution of CR8

CR8 was widely distributed into all tissues, and the time - concentration curves (AUCs) were fitted to one compartment for all tissues (Table 3).

**Table 3:** Pharmacokinetic parameters of CR8 in different organs following single oral administration of 100 mg/kg

	**Plasma**	**Liver**	**Adipose tissue**	**Kidney**	**Lung**	**Spleen**	**Bone marrow**	**Brain**
AUC (μg.hr/g)	96	197	181	145	77	40	34	14
Beta-HL (hr)	3.10	2.43	2.40	2.62	2.58	2.60	1.98	2.12
Cmax (μg/ml)	34.3	52.4	48.7	36.0	19.3	27.7	10.6	4.1
CL/F	21.0	10.2	11.1	13.8	26.2	20.0	29.5	146.0
V2/F	21.0	35.8	38.2	52.2	96.7	74.5	84.5	447.5

**Figure 6:**
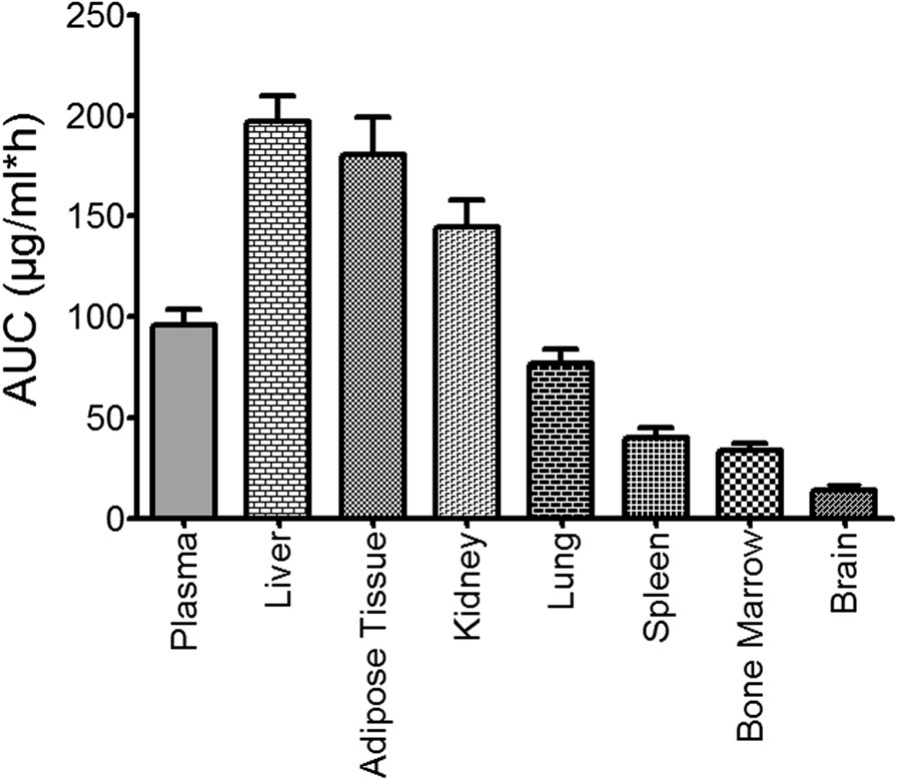
**Tissue exposure to CR8 after oral administration of 100 mg/kg to female BALB/c mice.** AUC expressed as μg/ml*h in plasma and as μg/g*h in tissues.

Higher exposure was found in the liver (the tissue/plasma exposure ratio was 200%), adipose tissue (180%) and kidneys (150%) compared to that observed as systemic exposure in plasma. Lung and spleen showed a tissue/plasma ratio of 80% and 50%, respectively (Figure 6) as compared to plasma.

#### CR8 distribution to the bone marrow

Exposure to CR8 was about 30% in bone marrow compared to in plasma when the concentrations were normalized to tissue weight/g (Figure 6).

This exposure is higher than that reported as IC_50_ required for the elimination of leukemic cell lines in about 6 h (data not shown).

#### CR8 distribution to the brain

CR8 was found to pass the blood–brain barrier (BBB) and the concentration in the brain was about 15% of that in plasma. Despite this low tissue/plasma ratio, the concentration reached a value above the reported CDK5/p25 and casein kinase 1 (CK1) inhibitory IC_50_ values for more than 6 hours.

## Discussion

Recently, the tri-substituted purine CR8 was introduced as a selective second generation CDK inhibitor analogue of roscovitine [6,7]. *In-vitro* studies have shown that CR8 is 2–4 fold more potent as a CDK inhibitor and 50–100 fold more cytotoxic in tumor cell lines [7]. However, this enhanced *in-vitro* activity of CR8 needs to be confirmed *in-vivo* in animal models prior to further development toward clinical trials in patients. The future investigation and development of this purine requires an accurate analytical method for the quantitative determination of its pharmacokinetics pattern. In the present investigation we have developed and validated a new analytical method for the determination of CR8 in plasma and tissue samples. Our data showed that the method is selective and accurate. The acceptance criteria for the method validation are well in accordance with internationally accepted criteria [15,18]. Moreover, the volume required for the analysis is only 50 μl of plasma, a favorable factor when considering plasma sampling from small laboratory animals.

The present method was employed for studying the pharmacokinetics and biodistribution of the drug in mice. It is of great importance to use a single, suitable analytical method for both pre-clinical and clinical studies of newly introduced drugs, such as CDK inhibitors, that are promising for the treatment of several diseases including cancer. One important factor is that the present method does not require work up procedures, a favorable factor for clinical studies where large numbers of samples are generated and speed of analysis is important.

The pharmacokinetics profile (especially the oral bioavailability and the elimination half-life) constitutes a set of critical factors, strongly influencing whether CR8 should enter preclinical and clinical trials as an anti-tumor agent. Good oral absorption and appropriate biological half-life allowing enough exposure to the drug to produce pharmacological activity and anti-tumor effect [22], and sufficient elimination allowing minimal toxicity, are indeed crucial for further development of an anti-cancer drug.

Roscovitine, which was introduced as one of the earliest CDK inhibitors, is at the present time undergoing phase II clinical trials in advanced non-small cell lung cancer and nasopharyngeal cancers [23]. Roscovitine was shown to have good oral bioavailability and low systemic toxicity profile [5,24]. However, the anti-tumor responses observed were modest, most probably a consequence of the drug’s short half-life and rapid metabolism to inactive metabolites [25]. Generally, exposure to the drugs is an important factor in the exertion of their anti-tumor activity. It has been shown that the optimum effect of roscovitine and the closely related analogue, N&N1, on tumor cell lines occurs after 8–16 hours of exposure [26-28]. The systemic exposure to roscovitine (expressed as AUC in plasma) in adult animals and humans did not allow a plasma concentration to be maintained above the reported anti-tumor IC_50_ (15–17 μM) for more than 1–2 hours, even when the maximum tolerated doses were administered [5]. The only exception was in young rats, most probably due to immature metabolism [29]. The present results showed that the systemic exposure expressed as plasma AUC of CR8 (after single dose administration of 100 mg/kg) was, for 10 hours, far higher than the anti-tumor IC_50_ values found *in-vitro* (0.3 μg/mL). Thus, second generation analogues of roscovitine might, in addition to increased cell potency, solve the problem of insufficient exposure observed in roscovitine. The higher systemic exposure might in part be explained by the longer half-life (approximately 3 h) found in female BALB/c mice in the present study. In this respect CR8 differs from both roscovitine [27,30] and its close analogue N&N1 [26], which both have a reported half-life of approximately 1 hour when given i.v. at the same dosage.

CR8 was found to have approximately 100% oral bioavailability, which means the drug is suitable for oral route intake and that the first pass effect is negligible.

CR8 distributed rapidly to all the peripheral tissues. The highest tissue distribution, as compared to plasma AUC, was found mainly in elimination organs, namely liver (200%) and kidneys (200%). These results encourage the hope that CR8 could perhaps be used to treat liver and kidney tumors, since it was found to induce apoptotic cell death in both hepatic and renal tumor cell lines [7]. Moreover, CR8 showed high distribution to the adipose tissue (180%) compared to that found in plasma, a factor that should be considered in dose scheduling in obese patients.

Surprisingly, CR8 showed higher distribution to the bone marrow (30%) compared to roscovitine, which was shown to pass the bone marrow barrier by only 1.5% [28] while the biodistribution of CR8 in the spleen was about 40%. Moreover, the cytotoxicity of CR8 against leukemic cell lines has been reported [7]. Altogether, these results suggest the possible use of CR8 in treating leukemia and lymphomas. However, the high biodistribution to the bone marrow may cause myelosuppression, which was not detected in roscovitine [28]. Further studies are currently ongoing in our laboratory to explore the cytotoxic effect of CR8 on normal hematopoietic progenitors. Moreover, we have observed five different metabolites with different polarities that have appeared with different kinetics throughout the CR8 kinetics. Urgent studies are needed to identify and quantify these metabolites, and to establish their pharmacological activates and toxicities.

In the present study, we have also shown that CR8 passes the BBB (concentration reached is 15% of that in plasma) compared to that reported for roscovitine (30%). This exposure of the brain to CR8 is nevertheless higher than that required for inhibition of its neuronal targets like CDK5 and CK1 (IC_50_ values: 0.12 μM and 0.61 μM, respectively) [7]. This fact may benefit the drug during further studies in neurodegenerative animal models and brain tumor models.

## Conclusions

We have developed and validated a simple, fast and selective quantitative analytical method which allowed us to analyze the pharmacokinetic profile and tissue distribution of the second generation CDK inhibitor CR8. CR8 was shown to have 100% bioavailability and longer half-life compared to roscovitine, which may allow higher systemic exposure than the IC_50_ values for tumor cell death induction. The drug passes the BBB at a level sufficient to reach concentrations able to inhibit neuronal kinase targets. Bone marrow exposure was about 30% of that found as systemic exposure, a concentration potentially sufficient to eliminate leukemic cells. However, further studies are required to determine the myelosuppressive effect and hematotoxicity of CR8. The present study thus shows favorable pharmacokinetics properties of CR8 compared to its parent analogue roscovitine, encouraging further *in-vivo* studies and consideration of CR8 as a drug candidate.

## Competing interests

The authors declare that they have no competing interests.

## Authors’ contributions

HS: carried out the experiments and drafted the manuscript. I ElS: performed the quality controls, and drafted the manuscript. LM: Provided the drug, drafted the manuscript. MH: Designed the experiment. All authors read and approved the final manuscript.

## Pre-publication history

The pre-publication history for this paper can be accessed here:

http://www.biomedcentral.com/2050-6511/14/50/prepub
